# Exploring gut microbiome and nutritional status among children with Autism Spectrum Disorder (MY-ASD Microbiome): A study protocol

**DOI:** 10.1371/journal.pone.0338801

**Published:** 2025-12-31

**Authors:** Kai Xuan Wong, Seong Ting Chen, Jun Jean Ong, Wan Ying Gan, Nor Azian Abdul Murad, Chun Wie Chong, Nurul Hanis Ramzi

**Affiliations:** 1 Centre of Postgraduate Studies by Research, IMU University, Kuala Lumpur, Malaysia; 2 Institute for Research, Development and Innovation (IRDI), IMU University, Kuala Lumpur, Malaysia; 3 Division of Nutrition and Dietetics, IMU University, Kuala Lumpur, Malaysia; 4 Paediatric Department, School of Medicine, IMU Clinical Campus Seremban, Negeri Sembilan, Malaysia; 5 Department of Nutrition, Faculty of Medicine and Health Sciences, Universiti Putra Malaysia, Serdang, Selangor, Malaysia; 6 UKM Medical Molecular Biology Institute (UMBI), Universiti Kebangsaan Malaysia (UKM), Cheras, Kuala Lumpur, Malaysia; 7 (FUTURE) Monash Microbiome Research Centre, Monash University Malaysia, Bandar Sunway, Selangor, Malaysia; 8 Centre for Cancer and Stem Cell Research, Institute for Research, Development and Innovation, IMU University, Kuala Lumpur, Malaysia; UCMI: University College MAIWP International, MALAYSIA

## Abstract

**Background:**

Autism Spectrum Disorder (ASD) is a neurodevelopmental disorder characterised by persistent deficits in social communication and the presence of restricted, repetitive behaviours or interests. Previous literature has identified a link between the gut and ASD; however, the underlying mechanisms remain unclear. Gut microbiota dysbiosis has been extensively reported in cohort studies of ASD, and specific microbial metabolites or by-products may serve as potential biomarkers for ASD. Additionally, children with ASD often exhibit food refusal, have a limited food repertoire and display a tendency to consume the same foods frequently; thus, these behaviours increase their risk of malnutrition (over-nutrition or under-nutrition) compared to typically developing (TD) healthy children. This study primarily aims to identify oral and gut microbiota among children with ASD and TD healthy children. The secondary aim is to determine the associations between oral and gut microbiota with nutritional status among children with ASD. The findings will enhance understanding of the aetiology of ASD and inform early intervention strategies to mitigate disease severity and early identification of malnutrition in genetically at-risk children.

**Methods and analysis:**

This observational, age-matched, case-control study is conducted in Malaysia among 40 male children with ASD and age-matched with 40 TD healthy controls aged 4–10 years. The dependent variables include the microbiota profile, identified through metagenomic sequencing analysis of saliva and faecal samples, and autism severity, assessed through validated questionnaires. Independent variables include nutritional status, determined through Subjective Global Nutrition Assessment (SGNA), anthropometry and dietary measurements, gastrointestinal symptoms, eating behaviour, behavioural profile, and sleep quality. Data collection is expected to be completed by June 2026. The study nature may limit causality establishment. Analyses will use chi-square/ANOVA for group comparisons, SparCC for microbiota correlations, and mixed-effects logistic regression to model associations.

**Conclusion:**

This study advances understanding of ASD-related microbiota, guiding personalised nutrition and precision healthcare in Malaysia.

## Background

Autism Spectrum Disorder (ASD) has both genetic and environmental origins [1]. Research has consistently shown that common and rare inherited variations (heritability) play a role in the genetic origins of ASD [1]. A multinational cohort study estimated the heritability of ASD to be approximately 80%, with modest differences in the sources of ASD risk across children from Denmark, Finland, Sweden, Israel, and Western Australia [1]. Moreover, the UK population-based twin cohort study reported ASD heritability estimates ranging from approximately 56% to 95% [2]. Nevertheless, debates persist regarding the relative importance of environmental factors compared to genetic predisposition in ASD [3]. Shared environmental effects refer to environmental factors common to individuals within a family or community, such as parenting practices, social and cultural influences, and exposure to toxins or pollution [4]. Recent research has identified that shared environmental effects can account for up to 50% of the variance in ASD risk in twin studies [5,6]. Furthermore, a recent meta-analysis research highlighted specific environmental factors that may contribute to ASD development, including prenatal exposure to toxins such as pesticides and air pollution, maternal stress during pregnancy, and early childhood experiences such as poor nutrition, neglect, and abuse [7]. These findings suggest that shared environmental factors may hold comparable significance to genetic factors in the development of ASD.

Children with ASD are frequently associated with disruptive mealtime behaviours, such as severe food refusal, a limited food repertoire, and high-frequency single-food intake [8]. These behaviours are closely related to sensory processing difficulties, such as aversion to specific textures, smells, and tastes [9]. Consequently, children with ASD are at an elevated risk of poor nutritional status. A local study conducted among 224 children with ASD in Kuala Lumpur reported that the prevalence of underweight, stunting, wasting/thinness and overweight/obesity were 9.3%, 8.0%, 4.0%, and 21.5%, respectively [10]. Another recent study in central region of Malaysia (Selangor, Kuala Lumpur, and Negeri Sembilan) involving a total sample size of 150 children with ASD documented that 15% of the children were underweight, and 52% were overweight and obese [11]. Although most children had adequate total energy and protein intakes, their fat intake exceeded the recommended levels [10]. Moreover, the majority of the children did not meet the Recommended Nutrient Intake (RNI) requirements for several key nutrients, including fibre (99.6%), thiamine (67.4%), vitamin C (50.9%), vitamin D (98.2%), vitamin E (74.6%), vitamin B12 (64.3%), folate (88.8%), calcium (90.2%), and zinc (77.2%) [10]. Therefore, a comprehensive understanding of the nutritional risks faced by children with ASD is essential for healthcare professionals to develop effective, targeted nutritional interventions.

Research has shown that children with ASD may exhibit altered nutrient metabolism, including deficiencies in essentials vitamins and minerals, which could contribute to the development and progression of the disorder. For example, studies have reported lower levels of vitamins B6 and B12 in children with ASD, both of are critical for neurological function [12–14]. Dietary habits are considered one of the main factors influencing the diversity of human gut microbiota [15]. Furthermore, host genetics, dietary habits, gut physiology, hygiene level, stress, and physical activity influence the gut microbiota. The human gut “metagenome” is a complex consortium of trillions of microbes, whose collective genomes contain over 100 times as many genes as the human eukaryote genome [16]. This essential “organ,” the microbiome, provides the host with protection against pathogens, enhancement of immune system and metabolic capabilities, as well as modulation of gastrointestinal (GI) development [17].

### Review of previous literature

The gut-brain axis is bidirectional, with signals from the brain also influencing gastrointestinal physiology and microbial composition through immune, endocrine and neural pathways [18,19]. Emerging evidence also suggested that the gut microbiome may play a role in the development and function of the brain, with some studies suggesting that alterations in the microbiome may contribute to ASD symptoms. For example, gut bacteria are capable of producing neurotransmitters such as serotonin and dopamine, which are critical regulators of mood and behaviour [20–22]. While some studies have reported consistent differences in the faecal microbiota of children with ASD [23,24], others have found no significant differences or have presented conflicting results [25]. The alterations in microbial composition have been linked to functional changes in brain activity and ASD-related symptoms, with studies implicating Clostridium overgrowth, inflammation, and disrupted neurotransmitter metabolism [18,19,26,29]. Studies have found that children with ASD have lower levels of *Bifidobacterium* and *Lactobacillus*, bacteria typically considered beneficial, and higher levels of *Clostridia* and *Desulfovibrio*, which are often associated with inflammation and gastrointestinal disturbances [26–29].

These findings have led to speculation that imbalances in the gut microbiome could contribute to the development or severity of ASD, potentially through mechanisms involving inflammation, immune dysregulation, or altered communication between the gut and brain (the gut-brain axis). While the relationship between nutrition, the microbiome, and ASD is still not fully understood, there is growing evidence suggesting that interventions targeting these factors may be beneficial in the managing ASD symptoms.

This study aims to address the knowledge gap by exploring the potential association between microbiota profiles, nutritional status and autism severity in children with ASD. Previous research has highlighted the potential role of gut microbiota imbalances, nutritional deficiencies, and their contribution to ASD progression [10,12–14,18,19,26–29]. However, there remains a lack of understanding of these relationships in Southeast Asia, particularly in Malaysia, where cultural and dietary practices may influence these factors differently from Western populations. This research is among the first comprehensive studies in Malaysia to simultaneously examine microbiota profile alongside nutritional status, particularly BMI and ASD severity. Existing studies have not fully integrated these components, often focusing solely on microbial diversity or gastrointestinal symptoms. The inclusion of nutritional status and a thorough analysis of dietary factors influenced by Malaysian culture are strengths of this study. The main strength of this research is its case-control study design, enabling the identification of specific microbiota and nutritional patterns associated with ASD severity as compared to TD healthy controls. By providing baseline data specific to the Malaysian context, this research aims to address the limited studies on the nutritional and microbiome profiles of children with ASD in Southeast Asia. With additional independent variables such as eating behaviour, behavioural profile, and sleep quality, the findings from this study could guide the development of targeted interventions, such as dietary modification or medical management, tailored to the unique needs of Malaysian children with ASD, ultimately enhancing their health and well-being.

### Objectives

This study primarily aims to understand the role of oral and gut microbiota in the molecular mechanisms underlying the aetiology of ASD among children in Malaysia. Specifically, this study aims to determine whether there is an association between the oral and gut microbiota and socio-demographic factors, nutritional status, and autism severity among children with ASD compared to TD healthy children. Additionally, this study also measures additional independent variables to determine the associations between oral and gut microbiota and gastrointestinal symptoms, eating behaviour, behavioural profiles and sleep quality.

**Fig 1:**
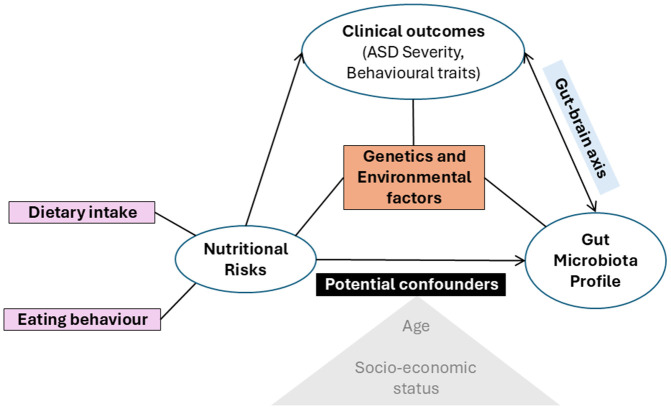
Conceptual framework.

The hypothesized relationships between gut microbiota, nutritional status, dietary intake, and ASD severity are illustrated in Fig 1.

## Methods and analysis

### Study design

This study employs an observational, age-matched case-control design, comparing children with ASD as cases with TD children as healthy controls. The case-control approach allows the identification of potential links and exposures related to risk factors for ASD. This design contributes to the growing body of evidence exploring the risk factors for ASD in children [30]. This study is currently in progress, with data collection and analysis expected to be completed in June 2026.

### Study Population and setting

Male children between 4–10 years old who comply with the protocol definition and inclusion criteria will be enrolled as participants. Cases are defined as male children diagnosed with ASD and receiving under follow-up care at the Paediatric Clinic at HTJ. Controls are defined as age-matched, TD healthy male children from daycare centres near IMU Healthcare and TABIKA KEMAS in Seremban. These locations were selected based on convenience sampling. Cases and controls are matched within using an age range of ±6 months.

### Sample size

This study aims to compare the microbial community structure between the ASD and TD healthy groups and within the groups, generating plausible distances relevant to the study objectives. Beta diversity is used to estimate the sample size or statistical power for this study. The recommended sample size is 60 participants, determined based on calculations using data from a study conducted by Li et al. [31]. Due to the limited availability of similar studies, this prior research served as a reference to obtain appropriate variance estimates [31]. The total study sample size of 60 participants has been adjusted to account for a possible 30% dropout rate; thus, this study aims to recruit 80 participants (40 children with ASD and 40 TD healthy children). The primary outcomes are microbial beta diversity and nutritional status. Secondary microbiota comparisons will be adjusted for multiple testing using the Benjamini-Hochberg false discovery rate, and post-hoc power analyses will be conducted to assess adequacy for multivariate models.

The study included only male children to eliminate potential gender bias, as the male predominance in ASD has been well-documented in epidemiological studies and widely recognised in the field [32–34]. This approach also ensures greater homogeneity in the demographic and microbiome datasets, thereby enhancing the precision and reliability of the findings.

### Eligibility criteria and screening procedures

The ASD group is male children aged 4–10 years diagnosed with ASD according to the Diagnostic and Statistical Manual of Mental Disorders, Fifth Edition (DSM-5) criteria, based on the evaluations performed by the child development team [35]. ASD diagnosis is confirmed by a developmental paediatrician through evaluations of medical history and clinical symptoms. The inclusion criteria for the ASD group are as stated: (1) male children aged 4–10 years who meet the DSM-5 criteria for ASD; and (2) whose parents or legal authorized representative (LAR) is willing to provide informed consent on their behalf. The exclusion criteria for the ASD group are as stated: (1) children below age of 4 years and above 10 years old; or (2) children who do not meet the case definition; or (3) children who have are on medications such as Ritalin and antipsychotics, anti-inflammatory, antioxidant drugs, or antibiotics in the three months prior to sample collection.

The TD group included healthy male children aged 4–10 years, with no history of early developmental delay, learning difficulties, or any other neurological or psychiatric conditions. Participants must not have been on medications such as Ritalin, antipsychotics, anti-inflammatory, antioxidant drugs or antibiotics in the three months prior to sample collection. Children with first degree relatives diagnosed with neurological, psychiatric, or neurodevelopmental disorders are excluded. TD children are defined as those achieving age-appropriate developmental milestones across gross motor, fine motor, language, cognitive, social-emotional and behavioural development. In Malaysia, routine health examinations monitor infant and child growth and developmental progress. Children were classified as TD if parents reported no developmental concerns from healthcare providers and no learning difficulties noted by schoolteachers.

### Recruitment

**Fig 2:**
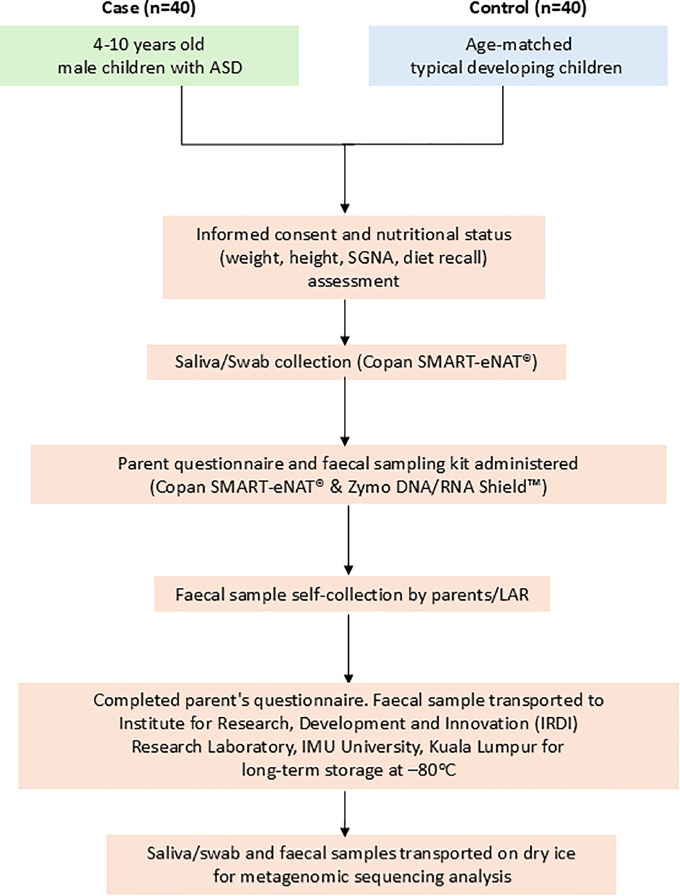
Flow diagram of study protocol workflow.

Children with ASD are identified by the developmental paediatrician at HTJ, Seremban, whereas TD healthy children are recruited from daycare centres or TABIKA KEMAS in Seremban. Data collection is performed by a trained graduate research assistant, acting as the research enumerator commenced on 24^th^ August 2024. Parents or legal guardians are given the study information sheet and to obtain their signed consent forms by the research enumerator. Participant recruitment and data collection are estimated to be completed in June 2026. Fig 2 below illustrated the study protocol workflow.

### Saliva and stool sample collection and analysis

Prior to saliva sample collection, participants will refrain from eating or drinking for at least 30 minutes, if this condition is not met, participants will be asked to gently rinse mouth with clean water before sampling. Saliva samples will be collected using 1 ml Copan eNAT^®^ swabs and 1 ml Copan SMART-eNAT^®^ (Cat. No. 70U001N, Copan Italia S.p.A., Brescia, Italy), both of which are guanidine-thiocyanate-based medium that inactivate microorganisms and stabilizes microbial DNA [36,37]. Two saliva samples will be collected on the day of recruitment and transferred to the laboratory at room temperature. Upon arrival, all the samples will be vortexed in the collection tubes until sample is homogenous, and divided into two aliquots as follows: 0.5 ml will be placed into four cryovials, respectively. All samples will be parafilm sealed, labelled with the collection date and specific study ID number (excluding personal information), and subsequently stored in a −80 °C freezer.

Stool samples will be collected using the 9 ml DNA/RNA Shield™ Faecal Collection Tube (Cat. No. R1101, Zymo Research, Irvine, CA, USA) [38] and 1 ml Copan SMART-eNAT^®^ (Cat. No. 70U001N, Copan Italia S.p.A., Brescia, Italy) [37]. Parents/LAR will receive a reminder via text message and a tutorial video outlining the timing and methods for self-collection of two faecal samples at home, along with instructions for storing the samples at room temperature. Once self-collection is completed, parents/LAR are required to notify the researchers to arrange transportation of the samples to the Institute for Research, Development, and Innovation (IRDI) research laboratory at IMU University, Kuala Lumpur, located approximately 50 km away. Faecal samples will be vortexed in the collection tubes until sample is homogenous, and the resulting stool slurry will be divided into two aliquots as follows: 0.5 ml will be placed into four cryovials, respectively. All samples will be immediately parafilm sealed, labelled with the collection date and specific study ID number (excluding personal information), and stored in a −80 °C freezer. All samples will be transported on dry ice to AGTC Genomics, Kuala Lumpur for further analysis.

DNA will be extracted using Zymo Quick-DNA MagBead Plus Kit. The purity of the extracted DNA samples will be assessed using Implen NanoQuant Spectrophotometer, and DNA libraries will be prepared using the DNA library Prep (Illumina, CA, USA) according to the manufacturer’s instructions. The prepared DNA libraries will be sequenced on Illumina Novaseq 6000 system (Illumina, CA, USA) at PE150. Libraries will be normalised to 1.75nM and pooled before loading into the NovaSeq sequencing platform. Demultiplexing of samples will be performed using Illumina DRAGEN BioIT Platform (Illumina) v3.9. CCMetagen pipeline will be used for taxonomic identification. Raw sequence reads (FASTQ files) will be trimmed and analysed using One Codex (https://www.onecodex.com/) to assess alpha diversity (as measured by the Shannon index) and to identify relative microbial abundances within the oral and gut microbiome of each participant.

The quality of raw reads will be examined with MultiQC [39]. Adapter sequences and low-quality reads (length < 50 bp or phred < 30) will be removed. To avoid potential human genome contamination, reads will be mapped against the hg38 human genome reference database (UCSC Genome Browser) using the Burrows–Wheeler Aligner (BWA) [40]. Unmapped reads (without human genome) will be used for downstream analyses. Bacterial taxonomic composition will be characterised using Kraken2 (v2.0.7, https://github.com/DerrickWood/kraken2) with the NCBI RefSeq database [41]. Kraken2, an ultrafast taxon-assigning tool, performs ultrafast taxonomic classification by aligning k-mers from the sequence data and assigning taxa based on the lowest common ancestor (LCA). Taxonomic abundances will then be re-estimated using Bracken (Bayesian Re-estimation of Abundance with KrakEN, v2.6), a Bayesian algorithm for recalculating species-level abundance [42]. HUMAnN3.5 (the HMP unified metabolic analysis network, https://github.com/biobakery/humann/tree/2.9) will be used to identify the functional genes/pathways associated with microbiome gene markers [43]. HUMAnN3.5 integrates with MetaPhlAn4 (Metagenomic Phylogenetic Analysis, v4, https://github.com/biobakery/MetaPhlAn4) for taxonomic profiling, utilizing its ChocoPhlAn pangenome database alongside the MetCyc, MinPath and UniRef90 databases [44]. From the HUMAnN2 output, gene family abundances with ≥90% match to UniRef90 will be used to convert this information to KEGG Orthologs (KOs: functional genes). Subsequently, the relative abundance of functional genes will be calculated based on the absolute abundance of all KOs for each sample.

### Data collection sheet

This sheet contains two sections as follows: (1) Information on participant and family demographics (child’s gender, age, gestational age, birth weight, parent’s age, ethnicity, occupation, education level, and household income); and (2) ASD medical history (age of first presentation, first presenting complaint, family history of ASD or other neurodevelopmental disorders (NDDs)) and other relevant medical conditions, if applicable.

### Anthropometric data

Stadiometer (SECA 213 Portable, Germany) and weighing scale (TANITA HD-319, Japan) will be used to obtain children’s height to the nearest 0.1 cm and weight to the nearest 0.1 kg. The BMI-for-age z score (BAZ), height-for-age z score (HAZ), and weight-for-age z score (WAZ) will be calculated using the WHO AnthroPlus Software version 1.0.4 [45]. BAZ, HAZ and WAZ classifications will be determined based on the WHO Child Growth Standards for children under five years of age and the WHO Growth Reference for children aged five years and above [46–48].

### Nutritional status and dietary assessment

A Subjective Global Nutrition Assessment (SGNA) will be utilised to determine the nutritional status of the children [49]. SGNA evaluates nutritional status in two main categories: Nutrition Focused Medical History (NFMH) and Physical Exam (PE). NFMH includes evaluating the appropriateness of current height and weight for age, changes in body weight, dietary adequacy, gastrointestinal symptoms, functional capacity, and metabolic aspects of disease. PE evaluates subcutaneous fat loss, muscle wasting, and nutrition-related oedema. The combined NFMH and PE evaluation will generate an overall SGNA ranking of normal/well-nourished, moderately malnourished, severely malnourished [49].

Dietary intake of the children will be obtained through parental interviews, using 3-day dietary recalls (two weekdays and one weekend day). To assist with the food intake portion size estimation, pictures of household measuring tools will be provided. Detailed information about the food and beverages consumed, such as cooking methods and brand names (if applicable), will be recorded. Nutrient analysis will be generated using the Nutritionist Pro^TM^ Diet Analysis software (Axxya Systems LLC, USA) and the Malaysian Food Composition Database (MyFCD). Nutrient adequacy will be assessed by comparing the intake to the Malaysian Recommended Nutrient Intakes (RNIs) [50]. Adequate intake will be defined as meeting or exceeding 100% of the RNI for macro- and micronutrients Portion size estimation in dietary assessment has been validated as reliable [51]. As dietary data will be parent-reported, research assistant will verify accuracy by reconfirming food intake during data entry prior to nutrient analysis. Basal metabolic rate (BMR) will also be calculated to identify potential underreporting or overreporting of energy intake.

Anthropometric measurements will be conducted by trained research assistant (WKX), a registered dietitian who completed standardized anthropometric assessment training measurement according to the standardized guideline by ISAK [52]. Observer and assessor training will be provided to research staff while obtaining data on 3-days dietary recall, with peer review and error checks to ensure data reliability.

### Parent report questionnaires

To analyse children’s eating behaviour, the Child’s Eating Behaviour Questionnaire (CEBQ), a standardized parent report questionnaire, will be used [53]. The CEBQ consists of 35 items, which are divided into eight subscales, which include food responsiveness (7 items), enjoyment of food (3 items), emotional overeating (3 items), desire to drink (3 items), satiety responsiveness (5 items), slowness in eating (4 items), emotional under-eating (4 items), and fussiness (6 items). Parents will rate the frequency of these behaviours using a 5-point scale: 1 representing never; 2 rarely; 3 sometimes; 4 often; and 5 always. The CEBQ subscale scores will be calculated by determining the mean score for each dimension, derived from the sum of the items divided by the number of items answered in each dimension. Higher scores reflect a higher intensity of the specific eating behaviour. For the five items with opposite phrasing, scores will be reversed according to the instrument’s instructions. CEBQ demonstrates good internal consistency, with Cronbach’s alpha ranging between 0.72 to 0.91, and adequate test-retest reliability (r = 0.52–0.87) [54]. The factorial validity of CEBQ has been evaluated in a study conducted on Dutch children, with Cronbach’s alpha ranging from 0.75 to 0.91, replicating the original eight-factor structure of the questionnaire and supporting its applicability across cultures [54]. Moreover, CEBQ has been validated among Malaysian children, showing good internal consistency within the subscales and widespread use in similar research [55].

Gastrointestinal (GI) symptoms will be assessed using a modified version of the GI Severity Index (6-GSI), which encompasses six items: constipation, diarrhoea, stool consistency, stool smell, flatulence, and abdominal pain [56]. Parents will rate their child’s GI symptoms on a three-point Likert scale, with scores of 0, 1 or 2 based on the severity and frequency of the symptoms experienced over the past three months. A score of 0 indicates the absence of symptoms, while scores of 1 and 2 reflect varying severity levels of the present symptom. A total score of 3 or higher will be considered a high score, while a score of below 3 will be considered as a low score [56]. The total GI score has a high intraclass correlation of 0.95 [95% CI: 0.87–0.98]. [57]

Furthermore, the Autism Treatment Evaluation Checklist (ATEC) is an instrument designed to quantitatively assess the effectiveness of treatments for children with ASD [58]. ATEC composes 77 items distributed across four subscales: 1) speech/language/communication, 2) sociability, 3) sensory/cognitive awareness, and 4) health/physical behaviour. The sum of the scores for each subscale gives the total ATEC score ranging from 0 to 179. For the interest of this study, only the first subscale, speech/language/communication (14 items) will be utilized to assess the language ability of children with ASD, with scores for this section ranging from 0 to 28. A lower total score indicates less severe symptoms of ASD, while a higher total score correlates with more severe symptoms of ASD. The internal reliability of ATEC is very high, with a total score reliability of 0.94, based on a split-half reliability test conducted on over 1,300 completed ATECs [59]. The results also showed good content validity and internal consistency (Cronbach’s alpha: 0.86–0.93) [59].

The Autism Quotient-Child (AQ) is a 50-item parent-report questionnaire using a Likert scale to assess autistic traits in children aged 4–11 years [60]. Parents will be asked to rate the extent to which they agree or disagree with statements about their child, using the following answer categories: 0 representing definitely agree; 1 slightly agree; 2 slightly disagree; and 3 definitely disagree. The Likert response format helps parents assess their children’s difficulty level, which is useful for screening for ASD and determining the severity of symptoms. The scale includes reverse items (1, 3, 8, 10,11, 14). Overall, the score range is from 0–150, with higher scores (76 and above) suggesting the presence of ASD traits. The sum of each item score represents the total AQ score, where a minimum AQ score of 0 indicates no autistic traits, and a maximum score of 150 suggests full endorsement of all autistic traits. The AQ-Child has been translated into multiple languages, with the Malay AQ-Child version demonstrating good reliability and validity with a Cronbach’s alpha value of 0.82 [61].

Moreover, the Strength and Difficulties Questionnaire (SDQ) is a 25-item parent-rating screening tool used to screen a child’s behavioural and emotional problems [62]. SDQ comprises five subscales: conduct problems (e.g., fighting with others, well-behaved), hyperactivity (e.g., restlessness, easily distracted), emotional symptoms (e.g., frequent worries, often unhappy), peer problems (e.g., being picked on or bullied, liked by other children), and prosocial behaviour (e.g., being considerate of others, shares with other children) [62]. Each subscale contains five items. Each item is rated on a 3-point response scale: 0 = not true, 1 = somewhat true, and 2 = certainly true. The scores from each domain will be summed, and a ‘total difficulties score’ will be calculated by summing the four deficit-focused subscales (excluding prosocial behaviour). Z-scores will be calculated for each domain, with higher Z-scores indicating fewer difficulties. The cut-off scores for ‘borderline’ and ‘abnormal’ classifications correspond to the 80th and 90th percentiles (20th and 10th for prosocial behaviour scale), respectively. Specifically, scores falling within the bottom 10% of the normative distribution indicate potential problems of clinical significance. The Malay version of parent-rated SDQ is readily available and has shown acceptable internal consistency, with Cronbach’s alpha values above 0.70 for all subscales, except for conduct and peer problems [63].

Children’s sleep quantity and quality will be assessed using two tools: the BEARS Sleep Screening Tool [64] and the Children’s Sleep Habit Questionnaire (CSHQ) [65]. BEARS is a brief, clinician-administered screening tool designed to identify common sleep issues in toddlers, preschoolers, and school-aged children. It includes five items and incorporates five basic sleep domains: Bedtime problems, Excessive daytime sleepiness, Awakenings during the night; Regularity of sleep/wake cycles and average sleep duration; and Snoring. These domains reflect the most common presenting sleep complaints in children. The clinical use of BEARS has been correlated with improved detection/diagnosis of sleep problems in children aged 2–12 years [64]. Besides, to assess sleep disorder scores among children with ASD, the 33-item abbreviated version of CSHQ will be used [65]. CSHQ is a more detailed parent-reported questionnaire that assesses sleep quality through eight subscales, each measuring specific types of sleep problems that will be summed up to a total sleep disorder score that indicated an index of sleep quality. Each item will be rated on a 3-point Likert scale, with items 1, 2, 3, 10, 11, and 26 being reversely scored. The total CSHQ scores range from 33 to 99. A high total sleep disorder score indicated a high sleep disturbance.

### Reimbursement

Participants are reimbursed a book voucher (RM50) as appreciation for their participation upon receipt of the stool sample and the parents’ questionnaire.

### Statistical analysis plan

Data analysis will be conducted using R software and its available packages. The differences in microbiota profiles, socio-demographic factors, emotional and eating behaviour, and nutritional status between children with ASD and TD children will be compared using the chi-square/Fisher’s exact test and ANOVA where appropriate. Student’s t-test will be applied to compare BMI and age between groups. Pre-filtering and inference of correlations will be performed using the Sparse Correlations for Compositional Data (SparCC) correlation metric [66], with a minimum R-value threshold of 0.35. SparCC will be used to correlate salivary and faecal bacterial markers detected in saliva and faecal samples from the same child. The associations between microbiota profiles, socio-demographic factors, emotional and eating behaviour, autism severity, sleep quality, gastrointestinal symptoms, and nutritional status among children with ASD will be modelled using mixed-effects logistic regression (MELR) model and random forest. Normality, linearity, homoscedasticity, and multi-collinearity will be assessed to ensure that the assumptions of regression models are met. Potential confounders, including age, and socioeconomic status will be adjusted for fixed effects. Model assumptions will be performed using DHARMa package in R to assess dispersion, zero-inflation and overall fit [67]. In case of heteroscedasticity or overdispersion, corrective strategies will include the use of an observation-level random effect, modelling dispersion or zero-inflation with glmmTMB [68], or applying bias-reduced logistic regression (Firth correction) [69] or bootstrap inference to mitigate small-sample bias [70]. Study results are expected in December 2026.

### Ethics approval and dissemination

This study has obtained ethics approval from the International Medical University Joint-Committee (IMU-JC) on Research & Ethics (Project ID: IMU R 299–2023) and the Medical Research & Ethics Committee (MREC) Ministry of Health (MOH) Malaysia (MREC ID: 23–03545-Q1W). Site approval has also been obtained from the Clinical Research Centre in HTJ, Seremban. Participation is voluntary, with written consent provided for the Parent Information Sheet (PIS), Informed Consent Form (ICF), ICF for future studies, Children’s Assent Form, and Children’s Assent for Biological Sample Collection. There are no severe side effects known to be caused by the assessment. This is an observational study with no interventions; parents are required to complete the questionnaires and faecal sample collection whereas investigators collected the child’s weight, height, and saliva sample, resulting in minimal risk for subjects. Subjects and their samples are linked to the study identification number, with no personal identifiers exposed. All personal and medical information obtained from the subject is kept in a password-protected database under the supervision of IMU University, Kuala Lumpur, Malaysia. Data and specimens are overseen by the MREC, MOH, and IMU-JC. Study results will be disseminated through peer-reviewed publications and conference presentations.

### Patient and public involvement

ASD patients are having their follow-up consultation with medical practitioners, eligible patients are invited to provide consent to take part in the study. ASD children’s parents are briefed on methods of self-collection stool samples. All other study outcomes are assessed by researcher and parents are not involved in the design, conduct, choice of outcome measures or the result dissemination of the study.

## Discussion

This study primarily aims to determine oral and gut microbiota profiles among children with ASD and investigate potential associations between the oral and gut microbiota and various factors, including socio-demographic factors, nutritional status, and autism severity among ASD and TD healthy children. This study also measures additional outcomes to determine the associations between oral and gut microbiota and gastrointestinal symptoms, eating behaviour, behavioural profile, and sleeping quality. This study addresses a critical knowledge gap within the Malaysian context, where unique cultural norms and dietary patterns may shape gut microbiome composition in ways that diverge from Western cohorts. These findings have the potential to inform precision, culturally tailored interventions for Malaysian children with ASD, advancing both clinical practice and public health strategies.

Where such bacterial genomic markers show an association with ASD, these could have an impactful effect on enhancing our ability to stratify patients for personalised therapy. This research will contribute to the increasing significance of genomic medicine within the Malaysian healthcare sector. This study could widen opportunities for applying structural genomic liability in both clinical and pharmaceutical contexts. By contributing to the essential fundamental knowledge of the genetics of ASD, this research could lead to advancements in personalized medicine for ASD treatment in Malaysia, benefiting the healthcare industry and ultimately improving the quality of care provided to affected individuals. This may result in a reduced economic burden of treating diseases that are the direct or indirect consequences of ASD in Malaysia. In alignment with national health priorities, this study embraces the Government’s efforts in fighting obesity and other diet-related non-communicable diseases (NCDs) via the National Strategic Plan for Non-Communicable Disease (NSP-NCD) and the National Plan of Action for Nutrition of Malaysia III (NPANM III).

The exclusion of children with ASD on medications is justified, as individuals with neuropsychiatric conditions often receive medications that can alter gut microbial communities [71]. Similar studies have also adopted this approach to minimize potential confounding effects on microbiota profiles in ASD [72]. This exclusion is therefore crucial to ensure an accurate assessment of the baseline microbial profile and better understand its relationship with ASD. Future intervention studies involving medicated children may instead aim to explore microbiota changes in response to specific pharmacological treatments.

The limitations of this study include its observational case-control design, reliance on parent-reported measures, and convenience sampling approach. Although observational in design, this study offers a critical foundation for future interventional research. The identified patterns may inform culturally tailored strategies such as probiotic supplementation or dietary modification to optimise child health in Malaysia. These applications, however, lie beyond the scope of the current protocol and require rigorous evaluation in longitudinal or randomised trials. Future longitudinal studies examining changes in the microbiome over time and in response to interventions could provide even more novel insights. Additionally, investigating serum indicators may help to optimise the evaluation of nutritional status.

## References

[1] Bai D, Yip BHK, Windham GC, Sourander A, Francis R, Yoffe R, et al. Association of genetic and environmental factors with Autism in a 5-country cohort. JAMA Psychiatry. 2019;76(10):1035–43. doi: 10.1001/jamapsychiatry.2019.1411 31314057 PMC6646998

[2] Colvert E, Tick B, McEwen F, Stewart C, Curran SR, Woodhouse E, et al. Heritability of Autism spectrum disorder in a UK population-based twin sample. JAMA Psychiatry. 2015;72(5):415–23. doi: 10.1001/jamapsychiatry.2014.3028 25738232 PMC4724890

[3] Tick B, Bolton P, Happé F, Rutter M, Rijsdijk F. Heritability of autism spectrum disorders: a meta-analysis of twin studies. J Child Psychol Psychiatry. 2016;57(5):585–95. doi: 10.1111/jcpp.12499 26709141 PMC4996332

[4] Hertz-Picciotto I, Schmidt RJ, Krakowiak P. Understanding environmental contributions to autism: Causal concepts and the state of science. Autism Res. 2018;11(4):554–86. doi: 10.1002/aur.1938 29573218

[5] Frazier TW, Thompson L, Youngstrom EA, Law P, Hardan AY, Eng C, et al. A twin study of heritable and shared environmental contributions to autism. J Autism Dev Disord. 2014;44(8):2013–25. doi: 10.1007/s10803-014-2081-2 24604525 PMC4104233

[6] Hallmayer J, Cleveland S, Torres A, Phillips J, Cohen B, Torigoe T, et al. Genetic heritability and shared environmental factors among twin pairs with autism. Arch Gen Psychiatry. 2011;68(11):1095–102. doi: 10.1001/archgenpsychiatry.2011.76 21727249 PMC4440679

[7] Emberti Gialloreti L, Mazzone L, Benvenuto A, Fasano A, Alcon AG, Kraneveld A, et al. Risk and protective environmental factors associated with autism spectrum disorder: evidence-based principles and recommendations. J Clin Med. 2019;8(2):217. doi: 10.3390/jcm8020217 30744008 PMC6406684

[8] Marí-Bauset S, Zazpe I, Mari-Sanchis A, Llopis-González A, Morales-Suárez-Varela M. Food selectivity in autism spectrum disorders: a systematic review. J Child Neurol. 2014;29(11):1554–61. doi: 10.1177/0883073813498821 24097852

[9] Stein LI, Lane CJ, Williams ME, Dawson ME, Polido JC, Cermak SA. Physiological and behavioral stress and anxiety in children with autism spectrum disorders during routine oral care. Biomed Res Int. 2014;2014:694876. doi: 10.1155/2014/694876 25114916 PMC4119730

[10] Eow SY, Gan WY, Awang H. Body weight status and dietary intake of Malaysian children with Autism Spectrum Disorder. Research in Autism Spectrum Disorders. 2021;84:101768. doi: 10.1016/j.rasd.2021.101768

[11] Zulkifli MN, Kadar M, Hamzaid NH. Weight Status and Associated Risk Factors of Mealtime Behaviours among Children with Autism Spectrum Disorder. Children (Basel). 2022;9(7):927. doi: 10.3390/children9070927 35883911 PMC9316127

[12] Adams JB, Audhya T, McDonough-Means S, Rubin RA, Quig D, Geis E, et al. Nutritional and metabolic status of children with autism vs. neurotypical children, and the association with autism severity. Nutr Metab (Lond). 2011;8(1):34. doi: 10.1186/1743-7075-8-34 21651783 PMC3135510

[13] Esteban-Figuerola P, Canals J, Fernández-Cao JC, Arija Val V. Differences in food consumption and nutritional intake between children with autism spectrum disorders and typically developing children: A meta-analysis. Autism. 2019;23(5):1079–95. doi: 10.1177/1362361318794179 30345784

[14] Mahruba SN, Begum S, Shahjadi S, Afroz S, Siddiqi UR, Parvin J. Serum vitamin B12 and folic acid status in Autism spectrum disorder children. J Bangladesh Soc Physiol. 2020;14(2):43–7. doi: 10.3329/jbsp.v14i2.44783

[15] Kashtanova DA, Popenko AS, Tkacheva ON, Tyakht AB, Alexeev DG, Boytsov SA. Association between the gut microbiota and diet: Fetal life, early childhood, and further life. Nutrition. 2016;32(6):620–7. doi: 10.1016/j.nut.2015.12.037 26946974

[16] Gill SR, Pop M, Deboy RT, Eckburg PB, Turnbaugh PJ, Samuel BS, et al. Metagenomic analysis of the human distal gut microbiome. Science. 2006;312(5778):1355–9. doi: 10.1126/science.1124234 16741115 PMC3027896

[17] Bäckhed F, Ley RE, Sonnenburg JL, Peterson DA, Gordon JI. Host-bacterial mutualism in the human intestine. Science. 2005;307(5717):1915–20. doi: 10.1126/science.1104816 15790844

[18] Fattorusso A, Di Genova L, Dell’Isola GB, Mencaroni E, Esposito S. Autism spectrum disorders and the gut microbiota. Nutrients. 2019;11(3):521. doi: 10.3390/nu11030521 30823414 PMC6471505

[19] Taniya MA, Chung H-J, Al Mamun A, Alam S, Aziz MA, Emon NU, et al. Role of gut microbiome in Autism spectrum disorder and its therapeutic regulation. Front Cell Infect Microbiol. 2022;12:915701. doi: 10.3389/fcimb.2022.915701 35937689 PMC9355470

[20] Strandwitz P. Neurotransmitter modulation by the gut microbiota. Brain Res. 2018;1693(Pt B):128–33. doi: 10.1016/j.brainres.2018.03.015 29903615 PMC6005194

[21] Dicks LMT. Gut bacteria and neurotransmitters. Microorganisms. 2022;10(9):1838. doi: 10.3390/microorganisms10091838 36144440 PMC9504309

[22] Wiley NC, Cryan JF, Dinan TG, Ross RP, Stanton C. Production of psychoactive metabolites by gut bacteria. Mod Trends Psychiatry. 2021;32:74–99. doi: 10.1159/000510419 34032647

[23] Korteniemi J, Karlsson L, Aatsinki A. Systematic review: Autism spectrum disorder and the gut microbiota. Acta Psychiatr Scand. 2023;148(3):242–54. doi: 10.1111/acps.13587 37395517

[24] Bezawada N, Phang TH, Hold GL, Hansen R. Autism Spectrum disorder and the gut microbiota in children: a systematic review. Ann Nutr Metab. 2020;76(1):16–29. doi: 10.1159/000505363 31982866

[25] Gondalia SV, Palombo EA, Knowles SR, Austin DW. Faecal microbiota of individuals with autism spectrum disorder. EJAP. 2010;6(2). doi: 10.7790/ejap.v6i2.213

[26] Argou-Cardozo I, Zeidán-Chuliá F. Clostridium bacteria and autism spectrum conditions: a systematic review and hypothetical contribution of environmental glyphosate levels. Med Sci (Basel). 2018;6(2):29. doi: 10.3390/medsci6020029 29617356 PMC6024569

[27] Mendive Dubourdieu P, Guerendiain M. Understanding the link between gut microbiota, dietary intake, and nutritional status in children with autism and typical development. Front Nutr. 2023;10:1202948. doi: 10.3389/fnut.2023.1202948 37545578 PMC10399235

[28] Heberling CA, Dhurjati PS, Sasser M. Hypothesis for a systems connectivity model of Autism Spectrum Disorder pathogenesis: links to gut bacteria, oxidative stress, and intestinal permeability. Med Hypotheses. 2013;80(3):264–70. doi: 10.1016/j.mehy.2012.11.044 23273906

[29] Doenyas C. Gut microbiota, inflammation, and probiotics on neural development in Autism Spectrum Disorder. Neuroscience. 2018;374:271–86. doi: 10.1016/j.neuroscience.2018.01.060 29427656

[30] Tenny S, Kerndt CC, Hoffman MR. Case Control Studies. Treasure Island (FL): StatPearls Publishing; 2024. https://www.ncbi.nlm.nih.gov/books/NBK448143/28846237

[31] Li N, Yang J, Zhang J, Liang C, Wang Y, Chen B, et al. Correlation of gut microbiome between ASD children and mothers and potential biomarkers for risk assessment. Genomics Proteomics Bioinformatics. 2019;17(1):26–38. doi: 10.1016/j.gpb.2019.01.002 31026579 PMC6520911

[32] Ben-Itzchak E, Ben-Shachar S, Zachor DA. Specific neurological phenotypes in autism spectrum disorders are associated with sex representation. Autism Res. 2013;6(6):596–604. doi: 10.1002/aur.1319 23873852

[33] Newschaffer CJ, Croen LA, Daniels J, Giarelli E, Grether JK, Levy SE, et al. The epidemiology of autism spectrum disorders. Annu Rev Public Health. 2007;28:235–58. doi: 10.1146/annurev.publhealth.28.021406.144007 17367287

[34] Holtmann M, Bölte S, Poustka F. Autism spectrum disorders: sex differences in autistic behaviour domains and coexisting psychopathology. Dev Med Child Neurol. 2007;49(5):361–6. doi: 10.1111/j.1469-8749.2007.00361.x 17489810

[35] American Psychiatric Association. Diagnostic and Statistical Manual of Mental Disorders, Fifth Edition. Arlington, VA: American Psychiatric Association; 2013.

[36] Copan Italia S.p.A. Product Focus eNAT®- Nucleic Acid Collection and Preservation Medium. 2024. [Cited 2024 October 14]. https://mediadelivery.copangroup.com/wp-content/uploads/2021/06/Product-Focus-eNAT.pdf

[37] Copan Italia SPA. Product focus SMART-eNAT® - Smart delivery system for nucleic acids preservation medium. 2024. [Cited 2024 October 14]. https://mediadelivery.copangroup.com/wp-content/uploads/2024/03/SMARTeNAT_JMKPF013R03.EN_DIGITAL.pdf

[38] Zymo Research Corp. Instructions for use- DNA/RNA Shield™ Faecal Collection Tube. 2023. [Cited 2024 October 14]. https://files.zymoresearch.com/quick-protocol/_r1101_dna-rna-shield-fecal-collection-tube.pdf

[39] Ewels P, Magnusson M, Lundin S, Käller M. MultiQC: summarize analysis results for multiple tools and samples in a single report. Bioinformatics. 2016;32(19):3047–8. doi: 10.1093/bioinformatics/btw354 27312411 PMC5039924

[40] Li H, Durbin R. Fast and accurate long-read alignment with Burrows-Wheeler transform. Bioinformatics. 2010;26(5):589–95. doi: 10.1093/bioinformatics/btp698 20080505 PMC2828108

[41] Wood DE, Lu J, Langmead B. Improved metagenomic analysis with Kraken 2. Genome Biol. 2019;20(1):257. doi: 10.1186/s13059-019-1891-0 31779668 PMC6883579

[42] Lu J, Breitwieser FP, Thielen P, Salzberg SL. Bracken: estimating species abundance in metagenomics data. PeerJ Comput Sci. 2017;3:e104. doi: 10.7717/peerj-cs.104 40271438 PMC12016282

[43] Beghini F, McIver LJ, Blanco-Míguez A, Dubois L, Asnicar F, Maharjan S, et al. Integrating taxonomic, functional, and strain-level profiling of diverse microbial communities with bioBakery 3. Elife. 2021;10:e65088. doi: 10.7554/eLife.65088 33944776 PMC8096432

[44] Franzosa EA, McIver LJ, Rahnavard G, Thompson LR, Schirmer M, Weingart G, et al. Species-level functional profiling of metagenomes and metatranscriptomes. Nat Methods. 2018;15(11):962–8. doi: 10.1038/s41592-018-0176-y 30377376 PMC6235447

[45] World Health Organization. Growth reference data for 5-19 years. 2019. [Cited 2024 October 9]. https://www.who.int/tools/growth-reference-data-for-5to19-years/application-tools

[46] World Health Organization. Body mass index-for-age (BMI-for-age). 2006. [Cited 2024 October 9]. https://www.who.int/toolkits/child-growth-standards/standards/body-mass-index-for-age-bmi-for-age

[47] World Health Organization. Length/height-for-age. 2006. [Cited 2024 October 9]. https://www.who.int/tools/child-growth-standards/standards/length-height-for-age

[48] World Health Organization. Weight-for-age. [Cited 2024 October 9]. 2006. https://www.who.int/tools/child-growth-standards/standards/weight-for-age

[49] Secker DJ, Jeejeebhoy KN. How to perform subjective global nutritional assessment in children. J Acad Nutr Diet. 2012;112(3):424-431.e6. doi: 10.1016/j.jada.2011.08.039 22717202

[50] National Coordinating Committee on Food and Nutrition. Recommended Nutrient Intakes for Malaysia. 2017. https://hq.moh.gov.my/nutrition/wp-content/uploads/2023/12/FA-Buku-RNI.pdf

[51] Amoutzopoulos B, Page P, Roberts C, Roe M, Cade J, Steer T, et al. Portion size estimation in dietary assessment: a systematic review of existing tools, their strengths and limitations. Nutr Rev. 2020;78(11):885–900. doi: 10.1093/nutrit/nuz107 31999347

[52] International Society for Advancement of Kinanthropometry. International Standards for Anthropometric Assessment. Potchefstroom, South Africa: International Society for the Advancement of Kinanthropometry; 2001.

[53] Wardle J, Guthrie CA, Sanderson S, Rapoport L. Development of the children’s eating behaviour questionnaire. J Child Psychol Psychiatry. 2001;42(7):963–70. doi: 10.1111/1469-7610.00792 11693591

[54] Sleddens EF, Kremers SP, Thijs C. The children’s eating behaviour questionnaire: factorial validity and association with Body Mass Index in Dutch children aged 6-7. Int J Behav Nutr Phys Act. 2008;5:49. doi: 10.1186/1479-5868-5-49 18937832 PMC2612017

[55] Tay CW, Chin YS, Lee ST, Khouw I, Poh BK, SEANUTS Malaysia Study Group. Association of eating behavior with nutritional status and body composition in primary school-aged children. Asia Pac J Public Health. 2016;28(5 Suppl):47S-58S. doi: 10.1177/1010539516651475 27252248

[56] Schneider CK, Melmed RD, Barstow LE, Enriquez FJ, Ranger-Moore J, Ostrem JA. Oral human immunoglobulin for children with autism and gastrointestinal dysfunction: a prospective, open-label study. J Autism Dev Disord. 2006;36(8):1053–64. doi: 10.1007/s10803-006-0141-y 16845577

[57] Thulasi V, Steer RA, Monteiro IM, Ming X. Overall severities of gastrointestinal symptoms in pediatric outpatients with and without autism spectrum disorder. Autism. 2019;23(2):524–30. doi: 10.1177/1362361318757564 29499612

[58] Rimland B, Edelson SM. Autism Treatment Evaluation Checklist. PsycTESTS Dataset. American Psychological Association (APA); 1999. doi: 10.1037/t03995-000

[59] Memari AH, Shayestehfar M, Mirfazeli F-S, Rashidi T, Ghanouni P, Hafizi S. Cross-cultural adaptation, reliability, and validity of the autism treatment evaluation checklist in persian. Iran J Pediatr. 2013;23(3):269–75. 23795248 PMC3684470

[60] Auyeung B, Baron-Cohen S, Wheelwright S, Allison C. The Autism Spectrum Quotient: Children’s Version (AQ-Child). J Autism Dev Disord. 2008;38(7):1230–40. doi: 10.1007/s10803-007-0504-z 18064550

[61] Hashmi SI, Ah Gang GC, Sombuling A, Md Nawi NH, Megat Ahmad PH. Psychometric properties and factor structure of the malay autism spectrum quotient: children’s version. Malays J Med Sci. 2021;28(6):108–20. doi: 10.21315/mjms2021.28.6.11 35002495 PMC8715883

[62] Goodman R. The strengths and difficulties questionnaire: a research note. J Child Psychol Psychiatry. 1997;38(5):581–6. doi: 10.1111/j.1469-7610.1997.tb01545.x 9255702

[63] Hassan NM, Husain R, Aziz AA, Mazubir NN, Juhari SN, Daud N. Validation of the malay version of the Self-rated Strength and Difficulties (SDQ) questionnaire. IJARBSS. 2019;9(11). doi: 10.6007/ijarbss/v9-i11/6553

[64] Owens JA, Dalzell V. Use of the “BEARS” sleep screening tool in a pediatric residents’ continuity clinic: a pilot study. Sleep Med. 2005;6(1):63–9. doi: 10.1016/j.sleep.2004.07.015 15680298

[65] Owens JA, Spirito A, McGuinn M. The Children’s Sleep Habits Questionnaire (CSHQ): Psychometric properties of a survey instrument for school-aged children. Sleep. 2000;23(8):1043–51. 11145319

[66] Friedman J, Alm EJ. Inferring correlation networks from genomic survey data. PLoS Comput Biol. 2012;8(9):e1002687. doi: 10.1371/journal.pcbi.1002687 23028285 PMC3447976

[67] Hartig F. DHARMa: Residual diagnostics for hierarchical (multi-level/mixed) regression models. 2022. https://cran.r-project.org/package=DHARMa

[68] Brooks ME, Kristensen K, Benthem KJ , van Magnusson A, Berg C W, Nielsen A, et al. glmmTMB balances speed and flexibility among packages for zero-inflated generalized linear mixed modeling. The R Journal. 2017;9(2):378. doi: 10.32614/rj-2017-066

[69] Firth D. Bias reduction of maximum likelihood estimates. Biometrika. 1993;80(1):27–38. doi: 10.1093/biomet/80.1.27

[70] Heinze G, Schemper M. A solution to the problem of separation in logistic regression. Stat Med. 2002;21(16):2409–19. doi: 10.1002/sim.1047 12210625

[71] Liu J, Gao Z, Liu C, Liu T, Gao J, Cai Y, et al. Alteration of gut microbiota: new strategy for treating autism spectrum disorder. Front Cell Dev Biol. 2022;10:792490. doi: 10.3389/fcell.2022.792490 35309933 PMC8929512

[72] Lewandowska-Pietruszka Z, Figlerowicz M, Mazur-Melewska K. Gut microbiota and autism spectrum disorders: neurodevelopmental, behavioral, and gastrointestinal interactions. Nutrients. 2025;17(17):2781. doi: 10.3390/nu17172781 40944170 PMC12430344

